# A resected case of pancreatic neuroendocrine neoplasm with completely cystic degeneration mimicking a mucinous cystic neoplasm: a case report and literature review

**DOI:** 10.1007/s12328-026-02309-0

**Published:** 2026-03-23

**Authors:** Tomohiro Arai, Naosuke Kuraoka, Risa Sunada, Keito Nakagawa, Morito Ikeda, Noriko Murakami, Akira Maki, Marino Nagata, Saburo Matsubara

**Affiliations:** 1 Department of Gastroenterology and Hepatology, Saitama Medical Center, Saitama Medical University, 1981, Kamoda, Kawagoe, Saitama, 350-8550 Japan; 2 Department of Hepatobiliary and Pancreatic Surgery, Saitama Medical Center, Saitama Medical University, Saitama, Japan; 3 Department of Pathology, Saitama Medical Center, Saitama Medical University, Saitama, Japan

**Keywords:** PNEN, Cystic degeneration, Pancreatic tumor, Pancreatic cyst, MCN

## Abstract

A 63-year-old woman was referred to our hospital because a cystic lesion was detected in the pancreatic tail on computed tomography (CT) performed at another hospital. Magnetic resonance cholangiopancreatography (MRCP) showed a 20 mm-sized unilocular cyst in the pancreatic tail; however no solid component was observed, then the patient was referred for follow-up observation. Six months later, MRCP showed an enlarged cyst, then contrast CT and endoscopic ultrasonography (EUS) were performed. Contrast-enhanced EUS showed a contrasted capsule and septum with internal hemorrhage. The patient was referred to surgery with a preoperative diagnosis of mucinous cystic neoplasm (MCN) with intracystic hemorrhage from a single, round, tail cyst, then robotic distal pancreatectomy was performed. Based on histopathological findings including immunostaining, the diagnosis of pancreatic neuroendocrine tumor (PNET G1) was made. Cystic neuroendocrine neoplasms of the pancreas are extremely rare, especially when they occur in the pancreatic tail as a cystic lesion mimicking an MCN, making preoperative differentiation extremely difficult. Such tumors should therefore be considered as a differential diagnosis from other cystic pancreatic tumors.

## Introduction

Pancreatic neuroendocrine neoplasms (PNEN) are relatively rare, accounting for less than 5% of all pancreatic tumors [1]. Recent advances in diagnostic imaging have increased the frequency of detection of small asymptomatic PNEN. PNEN is usually seen as a solid tumor, but cystic degeneration may also occur, and the cystic form is called cystic PNEN. In cases with a small amount of solid component, it is difficult to differentiate from the other pancreatic cystic lesions such as mucinous cystic neoplasms (MCN) or macrocystic variant of serous cystic neoplasms (SCN). In this report, we describe a case of cystic PNEN without solid components in the pancreatic tail that was preoperatively indistinguishable from MCN, with the review of the literature.

## Case report

A 63-year-old woman was referred to our department because a cystic lesion in the pancreatic tail was detected by cardiac computed tomography (CT) performed during an evaluation for palpitations.

The patient had a past medical history of hypertension and dyslipidemia. Her family history was unremarkable. She had no history of smoking and reported occasional alcohol consumption.

Abdominal magnetic resonance imaging/cholangiopancreatography (MRI/MRCP) showed a 20 mm unilocular cyst in the pancreatic tail. There was no solid component, and the patient was advised to follow-up. Six months later, MRI showed that the cystic lesion had increased to 35 mm in size, so additional examinations were performed. Blood test findings showed no elevation of pancreatic enzymes or tumor markers, and no worsening of HbA1c levels (Table 1).

**Table 1:** Laboratory data on the first visit

Parameter	Value	Unit
Hematology		
WBC	5.5	× 10^3^/μL
RBC	4.71	× 10^6^/μL
Hb	12.9	g/dL
Ht	40.3	%
Plt	32.7	× 10^4^/µL
Biochemistry		
Alb	4.0	g/dL
T-Bil	0.7	mg/dL
AST	17	U/L
ALT	17	U/L
LDH	190	U/L
ALP	85	U/L
γ-GTP	24	U/L
AMY	110	U/L
BUN	16	mg/dL
Cre	0.81	mg/dL
Na	145	mmol/L
K	3.8	mmol/L
Cl	106	mmol/L
Glucose	140	mg/dL
HbA1c (NGSP)	6.2	%
CRP	0.27	mg/dL
Tumor markers		
CEA	1.3	ng/mL
CA19-9	4	U/mL
DUPAN-2	36	U/mL
SPan-1	6.5	U/mL

**Fig. 1:**
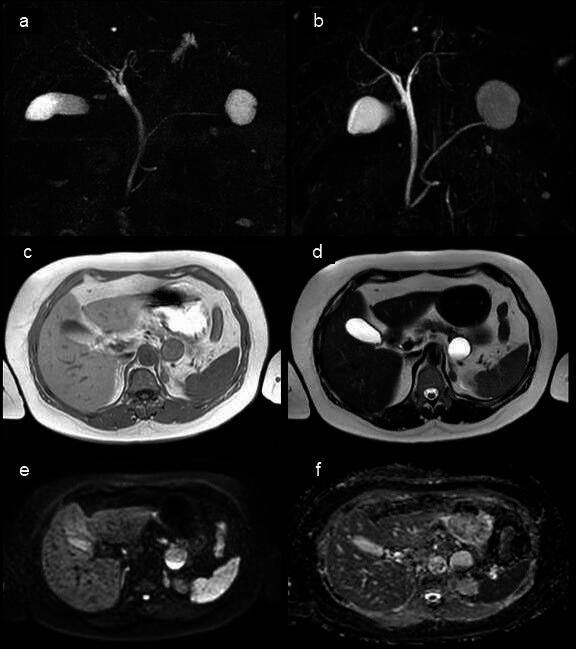
Abdominal magnetic resonance image/cholangiopancreatography (MRI/MRCP). **a** MRCP at initial examination. **b** MRCP after 6 months. **c** T1-weighted image. **d** T2-weighted image. **e** Diffusion-weighted image. **f** ADC-map. A 35 × 30 mm unilocular cyst was observed in the pancreatic tail. The dorsal surface of the cyst was fluid-forming and exhibited high signal on a diffusion-weighted image and low signal on an ADC map

MRI/MRCP showed a 35 × 30 mm unilocular cyst in the pancreatic tail  (Fig. 1). The interior of the cyst showed medium intensity on T1-weighted images (T1WI) and high intensity on T2-weighted images (T2WI). There was a fluid level formation on the dorsal surface of the cyst, high intensity on diffusion-weighted images and low intensity on apparent diffusion coefficient (ADC)-map, suggesting hemorrhagic changes.

**Fig. 2:**
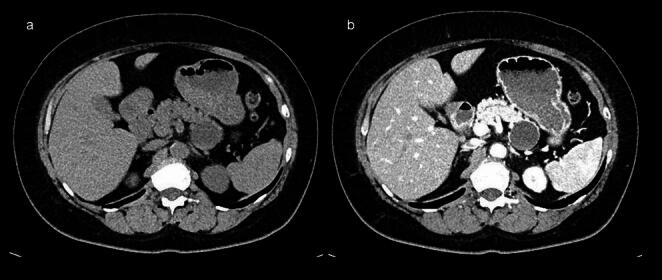
Abdominal computed tomography (CT). **a** non-enhanced CT. **b** Early phase of arteries on contrast-enhanced CT. A large, unilocular, 35-mm cystic mass was found in the pancreatic tail. Non-contrast CT showed mild hyperintensity within the mass

Contrast-enhanced CT showed a 35-mm-diameter, smooth-margined, unilocular cyst in the pancreatic tail with a thin, contrast-enhanced capsule. Non-contrasted CT showed slightly high-density lesion within the cyst, suggesting intra-cystic hemorrhage (Fig. 2).

**Fig. 3:**
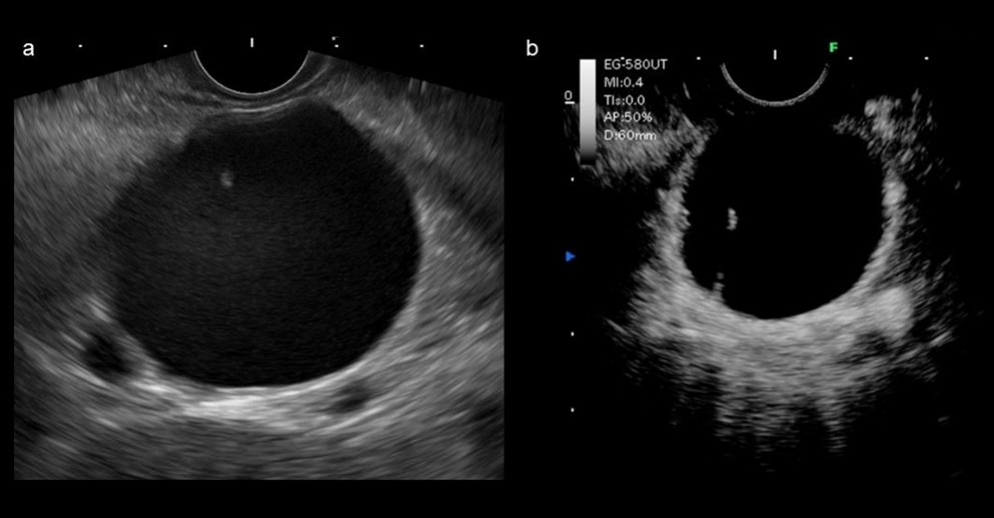
Endoscopic ultrasonography. **a** Endoscopic ultrasonography (EUS) showed a 37 × 30 mm round cyst in the pancreatic tail. A septate-like structure was observed within the cyst, and the cyst was filled with sludge echoes. **b** Contrast-enhanced EUS with perflubutane showed early contrast of the cyst wall

Endoscopic ultrasonography (EUS) showed a 37 × 30 mm round cyst in the pancreatic tail. A septate-like structure was observed within the cyst, and the cyst was filled with sludge echoes, suggesting hemorrhage (Fig. 3). EUS observation did not show any nodules in the wall. Moreover, the cyst wall was slightly thickened to 2 mm, and contrast-enhanced EUS (CE-EUS) with perflubutane showed early enhancement of the cyst wall. No main pancreatic duct dilatation was observed, and the connection between the cystic lesion and the main pancreatic duct was not observed.

**Fig. 4:**
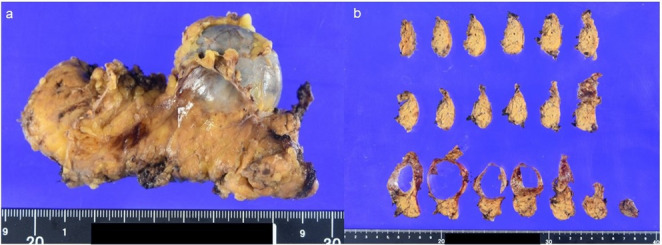
Gross image of the excised specimen. **a** A 43 × 40 × 38 mm cystic lesion was observed in the pancreatic tail. **b** The cyst wall was thin, and there was no obvious substantial component within the cyst

**Fig. 5:**
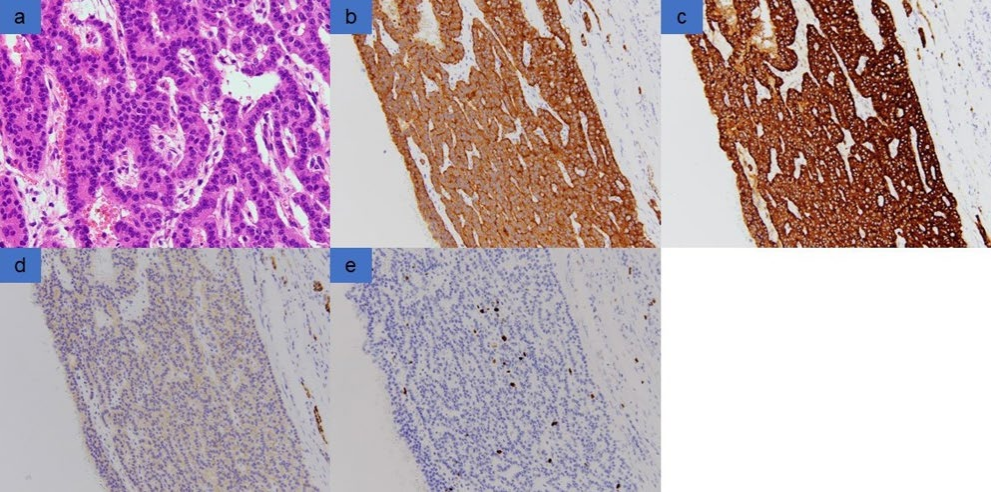
Pathological histological examination. **a** In hematoxylin and eosin (HE) staining, the cyst wall showed proliferating atypical cells with round nuclei and eosinophilic cytoplasm forming a reticular structure. **b**–**e** In immunohistochemical staining, the following were observed: synaptophysin (+) (**b**), chromogranin A (+) (**c**), CD56 (weak +) (**d**), and a Ki-67 labeling index of 2% (**e**)

Based on the above, we diagnosed MCN as a single, round pancreatic cystic lesion that occurred in the distal part of the pancreas in a middle-aged female. Robotic distal pancreatectomy was performed 10 months after the first visit. The resected specimen revealed a cystic lesion measuring 43 × 40 × 38 mm with a thin cyst wall and no obvious solid component (Fig. 4). The cyst was filled with serosanguinous fluid, and the septal-like structure observed on EUS was not observed. Histologically, the cyst was surrounded by a fibrous capsule, and the cyst wall showed proliferation of atypical cells with round nuclei and acidophilic cytoplasm arranged in a reticular pattern (Fig. 5). There was no increase of ovarian-like stromal cells in the subepithelial stroma. Immunostaining showed synaptophysin (+), chromogranin A (+), CD56 (weak +), and Ki-67 labeling index 2%, indicating PNET G1. The postoperative course was uneventful.

## Discussion

PNENs are relatively rare, accounting for less than 5% of all pancreatic tumors. However, their detection frequency has been increasing due to recent advances in diagnostic imaging modalities [1]. While typical PNENs present as homogeneous tumors with well-defined borders and strong contrast enhancement, the disease often exhibits variable imaging findings, including cystic degeneration, intraductal extension, and oligemia.

### Cystic PNENs: a distinct entity

Cystic PNENs represent a spectrum ranging from predominantly solid tumors with partial cystic degeneration to lesions where the cystic component occupies most of the tumor. These lesions occur in 10–17% of all PNENs [2], with pure cystic variants containing little or no solid component accounting for only approximately one-third of cystic cases [3]. Multiple mechanisms have been proposed for cystic transformation, including central necrosis or infarction associated with tumor growth and fibrous tissue formation, as well as intratumoral hemorrhage secondary to vascular disruption within these highly vascularized tumors [4].

In our case, the absence of necrosis within the tumor lumen and the presence of hematogenous fluid accumulation strongly suggest that intratumoral hemorrhage was the primary mechanism underlying cystic transformation. This finding supports the hemorrhagic etiology proposed in the literature.

### Clinical significance and malignant potential

Cystic PNENs, previously considered necrotic variants of solid PNENs, are now recognized as a distinct subgroup with significantly lower malignant potential. Singhi et al. analyzed 491 resected PNENs and found that 11% were cystic, demonstrating significantly lower rates of necrosis, invasion, metastasis, and Ki-67 index compared with their solid counterparts [5]. Histologically, these cysts typically present as unilocular lesions with thin fibrous capsules containing clear to pale yellow fluid.

### Diagnostic challenges

The diagnostic challenge becomes particularly pronounced when cystic PNENs present with minimal or absent solid components, especially when located in the pancreatic tail. In our case, the tumor exhibited a septal-like structure with complete cystic transformation, making preoperative differentiation from MCN extremely difficult. Such cases may be indistinguishable from MCNs, macrocystic variant of SCN, solid pseudopapillary neoplasm (SPN) with cystic degeneration, or epidermoid cyst in intrapancreatic accessory spleen (ECIPAS) based on imaging alone. These imaging findings and clinical characteristics are summarized in Table 2.

**Table 2:** Clinical and imaging features of pancreatic cystic lesions in differential diagnosis

Characteristics	Cystic PNEN	MCN	Macrocystic variant of SCN	SPN with cystic degeneration	ECIPAS
Age	50–60 years	40–50 years	60 years	20–40 years	40–50 years
Sex	No predilection	Almost exclusively female (> 98%)	Female predominance (60–80%)	Female predominance (90%)	Slight female predominance (57%)
Location	Throughout the pancreas	Body and tail	Throughout the pancreas	Body and tail	Tail
Capsule	Present	Present	Absent	Present	Present
Calcification	Possible	Possible	Possible	Possible	Extremely rare
Imaging features	On dynamic CT, the solid component shows homogeneous enhancement from the arterial phase with persistent enhancement	The capsule and septa are composed of fibrous connective tissue and show delayed enhancement on dynamic CT/MRI	High signal intensity on T2WI CT findings: central stellate scar and sun-burst appearance	On dynamic CT, the solid component shows mild enhancement in the arterial phase with gradual enhancement	On dynamic CT/MRI, the solid component demonstrates enhancement identical to that of the spleen across all contrast phases
Malignant potential	Present (mostly G1)	Present	Extremely rare (< 1%)	Present	Absent
Management	Resection based on size/grade; observation for small G1	Resection	Observation	Resection	Observation

*PNEN* pancreatic neuroendocrine neoplasm, *MCN* mucinous cystic neoplasm, *SCN* serous cystic neoplasm, *SPN* solid pseudopapillary neoplasm, *ECIPAS* epidermoid cysts in intrapancreatic accessory spleen

MCN typically presents as a unilocular or oligolocular cyst located in the pancreatic body or tail and predominantly affects middle-aged women. Characteristic features include a thick fibrous capsule, peripheral eggshell calcification, and the presence of ovarian-type stroma on histology. On contrast-enhanced imaging, MCN generally demonstrates enhancement of the cyst wall and septations, particularly on delayed-phase images [6, 7].

Macrocystic SCN may also mimic MCN or cystic PNEN, as it can present as an oligolocular cyst without the classic honeycomb appearance. Although uncommon, the presence of a central stellate scar with sun-burst appearance is pathognomonic. On MRI, SCN typically shows high intensity on T2WI with high ADC values, reflecting its serous fluid content [8].

SPN with cystic degeneration represents another important differential diagnosis, particularly because of its predilection for the pancreatic body and tail and its frequent association with intracystic hemorrhage. Although SPN classically occurs in young women, cases in older patients have been reported. Imaging features include a thick capsule, intralesional hemorrhage causing T1WI high intensity, and a gradual enhancement pattern [9].

ECIPAS should also be considered for cystic lesions in the pancreatic tail. This entity is particularly suggested when a solid component demonstrates enhancement identical to that of the spleen across all contrast phases. Identification of surrounding ectopic splenic tissue and confirmation using technetium-99 m–labeled heat-damaged red blood cell scintigraphy or superparamagnetic iron oxide–enhanced MRI are useful to establish the diagnosis and avoid unnecessary surgical intervention [10].

The diagnostic difficulty is reflected in the literature, with Singhi et al. reporting that 43% of cystic PNENs were not accurately diagnosed preoperatively [5]. In our case, the initial MRI revealed a small cystic lesion without solid components, leading to an initial diagnosis of benign or nonneoplastic cystic lesion and a decision for watchful waiting. With interval growth, the lesion was subsequently preoperatively diagnosed as MCN because it presented as a unilocular cyst in the pancreatic tail of a middle-aged woman without a definite mural nodule on EUS, while features suggestive of PNEN (hypervascular solid component), SPN (young age and thick capsule), SCN (honeycomb architecture or central scar), and ECIPAS (splenic-like enhancement) were absent.

While EUS is valuable for differential diagnosis of pancreatic cystic lesions, the absence of characteristic EUS findings specific to PNENs rendered preoperative diagnosis particularly challenging [11].

Fusaroli et al. demonstrated that CE-EUS is useful for identifying neoplastic features of pancreatic cystic lesions, emphasizing that enhancement of the cyst wall or mural structures is suggestive of neoplastic pathology even in the absence of a definite solid mass [12]. Therefore, cyst wall hyperenhancement on CE-EUS may represent an important diagnostic clue for cystic PNENs lacking an apparent solid component.

### Role of EUS-guided fine needle aspiration

EUS-guided fine needle aspiration (EUS-FNA) has become increasingly utilized for diagnosing pancreatic cystic lesions, with cytological or molecular analysis, tumor marker measurement, and through-the-needle procedures including probe-based confocal laser endomicroscopy or biopsy [13]. However, EUS-FNA for pancreatic cystic lesions carries inherent risks, including infection and potential tumor seeding [14]. Moreover, the diagnostic yield of EUS-FNA for cystic PNENs remains relatively modest (63.2–71%) [15].

We believe EUS-FNA should only be considered when a solid component can be safely targeted. In our case, the absence of a puncturable solid component precluded cystic sampling due to seeding risk. This conservative approach aligns with current best practices prioritizing patient safety.

## Clinical implications and future directions

This case highlights the importance of considering cystic PNEN in the differential diagnosis of pancreatic tail cysts, particularly in female patients presenting with septal structures or cyst-in-cyst morphology that may mimic MCN. Given the diagnostic challenges, a comprehensive imaging approach utilizing multiple modalities is essential to maximize diagnostic accuracy and guide appropriate management decisions.

The increasing recognition of cystic PNENs as a distinct entity with different biological behavior compared to solid PNENs has important implications for treatment planning and patient counseling. Future research should focus on identifying specific imaging biomarkers or molecular signatures that could improve preoperative diagnostic accuracy for these challenging lesions.

In conclusion, preoperative diagnosis of pure cystic PNENs remains challenging but should be included in the differential diagnosis of pancreatic cystic lesions, particularly when located in the pancreatic tail and presenting with complex cystic morphology. A multidisciplinary approach incorporating advanced imaging techniques and careful risk–benefit analysis of invasive diagnostic procedures is essential for optimal patient management.
